# Diagnostic and Therapeutic Management of *Helicobacter pylori* Infection in Primary Care: Perspective of Application in France and Narrative Review of the Literature

**DOI:** 10.3390/healthcare11030397

**Published:** 2023-01-30

**Authors:** Bernard Frèche, Julie Salvan, Marie Caroline Roch, Antoine Guerin, Elodie Poupin, Maxime Pichon, Christophe Burucoa

**Affiliations:** 1 Department of General Practice, Faculty of Medicine Pharmacy, University of Poitiers, 86021 Poitiers, France; 2 INSERM U1070, Faculty of Medicine Pharmacy, University of Poitiers, 86021 Poitiers, France; 3 Centre Hospitalier Universitaire (CHU) Poitiers, Bacteriology and Infection Control Laboratory, Infectious Agents Department, 86021 Poitiers, France

**Keywords:** *Helicobacter pylori*, disease management, infection control, primary care, stomach neoplasms

## Abstract

Background: *Helicobacter pylori* (*Hp*) infection affects 30% to 40% of people in industrialized countries. Aim: This study aimed to synthesize knowledge on the diagnostic and therapeutic management of *Hp* infection in general practice in people under 40 years of age. Method: A narrative review of the literature with an inductive content analysis of the articles was performed. Results: The extracted data (22 articles out of 106 included after screening of 965 articles) determined three areas of analysis: indications for screening, methods of screening and diagnosis by non-invasive tests, and treatment modalities. Discussion: Targeted, easily performed screening with noninvasive tests is recommended for patients younger than 45 years of age with no family history of gastric cancer and symptoms of dyspepsia without warning signs. Given their proximity to the general population and their coverage of the territory, general practitioners are ideally positioned. Treatment modalities are well-codified and feasible in primary care. Simplifying the recommendations available to them would optimize the identification of patients at risk and the management of *Hp* infection. Informing, educating, involving, supporting, and promoting the control of *Hp* infection in primary care will be future goals. Further research is needed in primary care to evaluate the impact of new procedures on *Hp* control.

## 1. Introduction

*Helicobacter pylori* (*Hp*) infection was first identified in the 1980s by Dr. Barry Marshall and Dr. Robin Warren in gastric biopsy samples from people with peptic ulcers [1]. It was not until the 1990s that the scientific community definitively accepted the link between *Hp* infection and peptic ulcers [2]. This infection affects two thirds of the population living in developing countries and about 30 to 40% of people living in an industrially developed country [3]. In France, it is estimated that in one year, the entire population consults a primary care physician [4]. Symptoms related to upper gastrointestinal pathology that may suggest *Hp* infection are recurrent reasons for consultation in general practice [5]. However, the presence of these symptoms is still strongly debated by the scientific community for indication of diagnosis and treatment of *Hp* infection among gastroenterologists, pediatricians, and general practitioners. In fact, the majority of *Hp* infections are asymptomatic [6]. Best practice guidelines emphasize the clinical consequences, symptoms, and syndromes of the infection as well as its treatment [7]. Gastric and duodenal inflammation as well as ulcerative disease are responsible for upper GI symptoms. The latter are present in other pathologies such as gastroesophageal reflux and functional intestinal disorders. *Hp* has been recognized by the World Health Organization (WHO) since 1994 as the only carcinogenic bacterium [8]. The highly oncogenic nature of certain genotypes of *Hp* creates a public health problem. Gastric cancer is the fourth most common cancer in men and the fifth in women. It has a poor prognosis and a 5-year survival rate under 20% [9]. This cancer was the cause of 800,000 deaths in 2020 worldwide according to WHO GLOBOCAN estimates [10]. In spite of this, French recommendations to date oppose systematic screening and advocate targeted screening based on risk factors. For example, screening for *Hp* is indicated in case of a history of ulcer or active ulcer, complicated or not, without previously documented *Hp*, chronic dyspepsia with long-term treatment with proton pump inhibitors (PPI) (>6 months), iron deficiency anemia with no known cause or resistant to oral iron treatment, and vitamin B12 deficiency with no etiology found. Also included are patients with gastric MALT lymphoma, patients requiring bariatric surgery isolating part of the stomach, and patients with adult immunologic thrombocytopenic purpura [11]. Finally, patients with risk factors for gastric cancer are obviously included: first-degree relatives of a patient who has had gastric cancer, patients with a predisposition syndrome for digestive cancers, patients who have undergone partial gastrectomy or endoscopic treatment of gastric cancer lesions, and patients with gastric preneoplastic lesions with severe atrophy and/or metaplasia and/or intestinal dysplasia. It is recommended that patients with a history of gastric or duodenal ulcers consider testing for *Hp* before taking nonsteroidal anti-inflammatory drugs or low-dose aspirin [12]. In 2017, the recommended diagnostic strategy in a country such as France targeted the search for *Hp* in these specific indications coupled with diagnostic, biological, or endoscopic means. Three non-invasive techniques are currently available: the urea breath test, stool testing for *Hp* antigens, and serology by ELISA [13]. Serology is recommended as a first-line test in patients under 40–45 years of age, first-degree relatives of patients who have had gastric cancer, or those with a history of ulcer or immunologic thrombocytopenic purpura. Negative serology is sufficient to rule out *Hp*. Positive serology is an indication for gastric endoscopy. Invasive techniques require endoscopy to perform biopsies for pathological analysis, PCR gene amplification, rapid urease testing, and culture. Nelson et al. in 2021 published a review of the literature to answer the question “who and how to test and treat using recommendations for first- and second-line therapies, eradication monitoring, and strategies for use in primary care” [14]. However, there exist discrepancies between national, European, and even global recommendations for screening, diagnosis, treatment, and control of eradication of *Hp* in adults under 40 years of age and the practice of primary care physicians and, to a lesser degree, gastroenterologists. This study aimed to synthesize knowledge about the diagnostic and therapeutic management of *Hp* infection in general practice in adult persons under 40 years of age.

## 2. Methods

For this purpose, a narrative review of the literature with inductive analysis of the contents of the articles was conducted. An exhaustive search of published and unpublished articles that answered our questions was carried out using the Web of Science, BDSP, and OpenGrey databases, the PubMed, Google Scholar, and Google search engines, and other resources such as the National Cancer Institute, the British Society of Gastroenterology, the United European Gastroenterology, the Société Nationale Française de Gastroentérologie, the National Institute for Health and Care Excellence, the British Society of Gastroenterology, the Top Alberta Doctors, and the Gastroenterological of Australia. The literature search was led by a member of the Department of Observation, Monitoring, and Evaluation of the Institut Nationale du Cancer (INCa), in English and French. The keywords used were “prevention”, “*Helicobacter pylori*”, “proton-pump inhibitor”, “ambulatory care”, “screening”, and “detection”. The search equations used are available in the appendix. A first selection was made according to the title and the abstract of the articles by five researchers: two university teachers of primary care, one teacher-researcher in bacteriology, and two students in the General Medicine thesis program. The bibliographic references of each article were explored secondarily. In case of disagreement between the reviewers, a consensus was reached after discussion. The search was conducted from 24/01/19 to 04/03/19. It included articles dating from 1982, the year of the discovery of *Hp*, to 31 December 2018. Original research articles, systematic literature reviews, grey literature, and any reports and expert opinions deemed relevant were included. Selected articles were read in full. Data extraction included: title, authors, date of publication, journal of publication, country of origin, methodologies or type of articles, objectives of the article, characteristics of the study population, primary outcomes, secondary outcomes, and conclusion(s). A standardized extraction grid was developed (Supplementary Tables S1 and S2).

**Figure 1:**
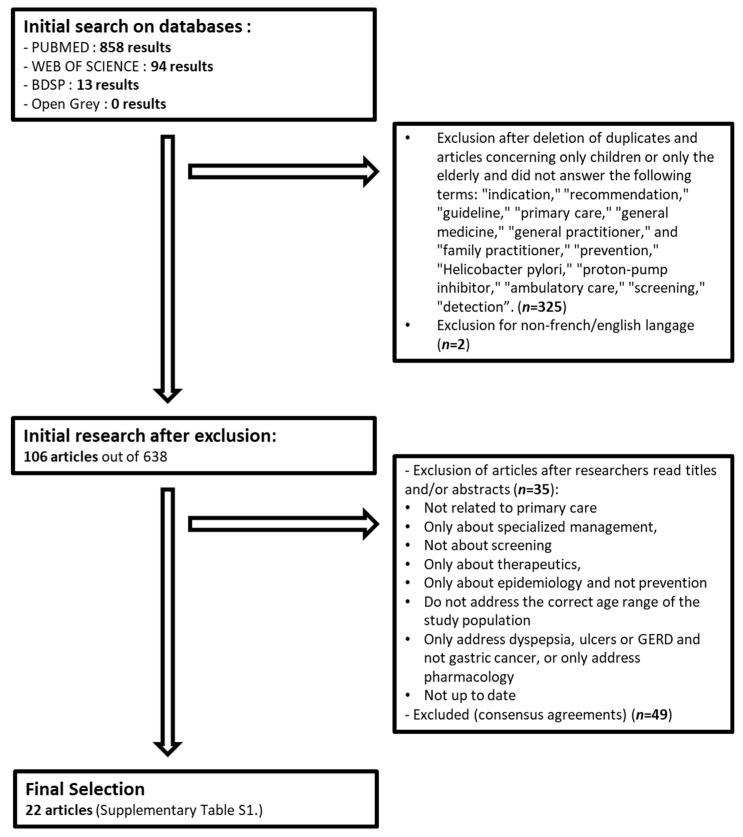
Flow chart for selection of analyzed articles.

As described in Figure 1, a total of 965 articles were identified. After deleting duplicates and articles concerning only children or the elderly, and after a relevance analysis had been performed by the documentarist, a total of 106 articles were included. After reading the titles and abstracts, 22 articles were read and analyzed in full. The results of this search were discussed under four main themes: indications for screening, methods of screening and diagnosis by non-invasive tests, and treatment modalities.

## 3. Results

### 3.1. Indications for Screening for Helicobacter pylori

In the context of epidemiological studies or personalized medicine, screening can be either global/systematic or focused/clinically guided depending on the situation.

#### 3.1.1. Systematic Screening

General population screening is still feasible in countries with a high prevalence of *Hp* infection (Asia, Africa, Central America, Eastern Europe), where the prevalence of *Hp* is about 50% [15,16,17]. In Asia Pacific, routine screening and treatment of *Hp* infection has been implemented in the high-risk population. It has been suggested that screening should begin 10 to 20 years before the age of highest incidence of gastric cancer [18]. In Taiwan, the *Hp* stool antigen kit was added to the stool blood test used for colorectal cancer screening to detect upper intestinal lesions, primarily due to *Hp*. The same tests for a flagellum antigen have been used to detect *Hp* in saliva in Chinese studies [19].

#### 3.1.2. Focused Screening

In countries with low prevalence of *Hp* infection (Europe, Canada, United States), population-based screening is not recommended. It is preferable to target preventive strategies to high-risk individuals through individual screening [18,20].

Indeed, general population screening would pose ethical problems due to the possible adverse effects and potential anxiety generated in apparently healthy individuals. Moreover, a mass eradication program would not be feasible from a practical and economic point of view, given the cost of an effective antibiotic treatment, the problems of compliance with the treatment, the undesirable effects, and the induced resistances [18,21].

Finally, the lack of long-term data and appropriate prospective studies make it impossible to consider screening for *Hp* in the general population [20,22].

As part of targeted screening for *Hp* infection, it is recommended that patients under 45 years of age with no family history of gastric cancer and/or symptoms of dyspepsia without warning signs such as anemia, weight loss, dysphagia, palpable mass, or malabsorption be screened in primary care. Complementarily, it is strongly recommended that patients over 45 years of age with symptoms of dyspepsia, and those with warning signs regardless of age, be referred to gastroenterologists for a histological study in search of pre-neoplastic or neoplastic lesions [7,15,23,24].

### 3.2. Screening and Diagnostic Methods: Non-Invasive Tests

*Hp* infection can be detected by both invasive and noninvasive methods. Endoscopy and biopsies are not feasible for general population screening [25]. Therefore, screening can be performed by urea breath test, serology, and stool antigen detection in the primary care setting [15].

#### 3.2.1. Urea Breath Test

The urea breath test is the non-invasive test of choice for the diagnosis of active *Hp* infection and monitoring of eradication after treatment [15,23,26,27]. In primary care, it is widely used in dyspeptic patients without warning signs [25,27]. Sensitivity and specificity are high (>95%), provided that PPIs are stopped two weeks (and antibiotics four weeks) before the examination [15,23,24]. However, it has two disadvantages: i/duration of at least 30 min; ii/price, although it is reimbursed in France for the initial diagnosis and eradication control [24,28]. 

#### 3.2.2. Serology

Screening for *Hp* infection by serological testing is available in the overall context of initial diagnosis [15]. As IgG antibodies are present for a long time in the body after infection, serology does not allow dating of the infestation. That is why it is not indicated for post-treatment eradication control [15,23,24,26,29].

#### 3.2.3. *Hp* Antigens in Stools

*Hp* antigen detection testing in stool is the other non-invasive test to detect active *Hp* infection and monitor post-treatment eradication [24]. Sensitivity and specificity are similar to those of the urea breath test, so that it can be used as an alternative [15,19,23]. This test requires the same precautions as the urea breath test regarding treatment (stopping PPIs two weeks before and stopping antibiotics four weeks before) [15,23,24]. Economically, it is less expensive and requires less equipment than the urea breath test [24]. Due to the prolonged shedding of *Hp* antigen, it is recommended that confirmation of treatment be performed up to six weeks after the end of treatment [23]. Note that it as of now, it is reimbursed in France.

### 3.3. Treatment Modalities

After successful diagnosis of *Hp* infection, it is necessary to plan treatment in order to effectively eradicate this risk factor for tumorigenesis.

The first effective treatment for *Hp* was introduced at the end of the 1980s, using a combination of bismuth salts, tetracycline, and metronidazole for 14 days. However, the side effects were numerous and compliance was incomplete [29].

PPI utilization improved the eradication rate and led to a new and better tolerated treatment, the triple therapy combining PPIs, clarithromycin, and amoxicillin for 7 to 10 days, which for 25 years remained the reference treatment [7,21,26,30].

The last decade was characterized by a marked decline in the success of triple therapy due to the emergence of resistance to clarithromycin [24].

Resistance rates to metronidazole, clarithromycin, and more recently, levofloxacin have reached 30% worldwide. In contrast, resistance to amoxicillin and tetracycline remains rare (less than 2%) [15].

In the USA, the choice of treatment is made according to the rate of resistance, the cost of the molecules available, and allergy (or not) to penicillin [15,24]. The recommended first-line treatment is four-drug therapy with bismuth salts for 14 days in case of high resistance to clarithromycin. In case of low prevalence of primary clarithromycin resistance (<15%), triple therapy with clarithromycin, PPI, and amoxicillin for 14 days is recommended. In case of allergy to bismuth salts, quadruple therapy with clarithromycin, PPI, amoxicillin, and metronidazole is recommended. In case of failure, second-line treatment is either with quadruple therapy with bismuth if clarithromycin is used initially, or with concurrent quadruple therapy. Levofloxacin can also be used, but a lack of relevant studies and a lack of hindsight do not make it a treatment of first choice [15,24].

In Europe and Asia, quadruple therapy appears to be superior to triple therapy, but this treatment has not yet been sufficiently studied in the USA [24].

According to the latest (May 2017) French HAS recommendations, in the absence of antibiotic susceptibility studies, treatment with concomitant quadruple therapy is recommended as first-line therapy, combining a PPI, amoxicillin, clarithromycin, and metronidazole for 14 days. In case of previous use of macrolide or allergy to amoxicillin, quadruple therapy with bismuth for 10 days is preferred, combining omeprazole with a bismuth salt, tetracycline, and metronidazole. Concomitant quadruple therapy replaces sequential treatment because of its better efficacy, especially on clarithromycin-resistant strains of *Hp* [31].

## 4. Discussion

General practitioners represent the first line of care in the management of *Hp* infection. Two studies in Israel have been conducted to assess the knowledge and practices of general practitioners [16,23]. The indication to screen patients with peptic ulcer for *Hp* is met 95% of the time by general practitioners and specialists [32]. However, only 25–30% of general practitioners screen first-degree relatives of patients who have had gastric cancer for *Hp* [16,17,33]. In addition, only 14% of general practitioners look for *Hp* in patients who are going to be treated with long-term NSAIDs despite the recommendations [16]. On the other hand, one third of general practitioners are likely to screen for *Hp* in patients with active GERD symptomatology, which is inconsistent with the recommendations [16,33]. For diagnostic methods, 60 to 97% of general practitioners prefer the urea breath test [16,33]. However, only 40% stop the necessary treatments 14 days before performing the breath test, as recommended. Only 2–5% use the stool antigen test, the other gold standard [16,33]. Regarding good treatment practices, triple therapy with amoxicillin, clarithromycin, and PPI is by far the most commonly used first-line treatment by general practitioners (75–85%). However, in 70% of cases, the duration of treatment is less than 14 days [16,33]. In Israel, 40% of general practitioners prescribe concomitant therapy as a second line of treatment and 30% would prescribe bismuth quadruple therapy [16]. Finally, the results showed that less than half of general practitioners confirm eradication of infection by a non-invasive test (16–40%) [16,33]. The contrast in management by primary care physicians around the world may reflect differences in health care systems, epidemiology, and approaches to the management of *Hp* infection in different regions [22]. 

Most recommendations were written by medical specialists to harmonize the management of *Hp* infection. Primary care practitioners agreed to follow these recommendations [22]. The best known are the Maastricht recommendations, the first of which began in 1997. In 1999, the impact of the Maastricht recommendations was evaluated in many European countries by interviewing general practitioners and specialists. It was shown that about 50% of physicians were aware of the existence of the Maastricht guidelines. Primary care practitioners generally agree to abide by these recommendations and would agree that information and recommendations regarding the management of *Hp* are useful to their practice [7]. However, practices in primary care remain heterogeneous. Two studies in Israel have shown that there is a variation in implementation of the 2000 Maastricht recommendations between specialists and general practitioners. The 2004 study by Shirin showed that the 2000 Maastricht recommendations were better applied by gastroenterologists and internists than by general practitioners, particularly with regard to the importance of diagnosing and eradicating *Hp* in cases of malignant gastric lesions or NSAID use [33]. The other 2016 study conducted by Boltin highlighted a disparity in practices by general practitioners, with better application of the recommendations in city centers than in rural areas [16]. This heterogeneity of practices could be reduced by the implementation of information campaigns, which could target the indications for *Hp* screening, the practical procedures for carrying out non-invasive tests, and the updating of first and second-line treatments according to the specific epidemiology of the region [16]. General practitioners would like to receive more educational programs on the management of *Hp* infection in order to conserve health resources and increase the efficiency of patient management [16,25,33]. In fact, insufficient follow-up of these recommendations in primary care tends to impact the effectiveness of the prescribed treatments, with increased clarithromycin resistance over the last 10 years and decreased eradication of *Hp*. For example, the 2016 Israeli study by Boltin showed that 38% of GPs would use concomitant therapy and 6% would then prescribe the same treatment if it failed. Furthermore, most prescribers of triple therapy with amoxicillin, clarithromycin, and PPI prescribe the treatment for less than 14 days, despite recommendations. However, in 2013, a Cochrane meta-analysis of 75 randomized clinical trials found that extending clarithromycin triple therapy from 7 to 14 days significantly increased the *Hp* eradication rate (72 to 81.9%) and decreased the risk of treatment failure (RR 0.66, CI95 0.60–0.74) This lack of implementation of recommendations leads to increased bacterial resistance and cost of care [16].

European recommendations recommend that a specialist be consulted after the failure of two lines of treatment in order to determine the source of resistance of *Hp* to antibiotics by performing an antimicrobial susceptibility testing [24].

Screening for *Hp* infection is necessary for the prevention of gastric cancer. In countries with a low prevalence of *Hp*, population-based screening is not recommended. Targeted screening is recommended for patients under 45 years of age with no family history of gastric cancer and presenting with symptoms of dyspepsia without warning signs. This type of individual screening is feasible in primary care, especially since general practitioners are regularly and predominantly confronted with this target population. On the other hand, it is recommended to refer to gastroenterologists, patients over 45 years of age with symptoms of dyspepsia, and those with warning signs regardless of age, the objective being to detect pre-neoplastic lesions. The first-generation serological tests had lower sensitivity and specificity than the other methods, around 79–85% [15,23,24]. Second-generation serological tests currently have good performance (sensitivity and specificity greater than 90%), as demonstrated in the study by Burucoa et al. published in *Helicobacter* in June 2013 [32]. Since this article, the authors have recommended serology for diagnosis and the Haute Autorité de Santé (HAS) in France has integrated it into its diagnostic algorithm. The advantages of this test are the low cost and the absence of alteration by PPI or antibiotics [7,15,24]. 

Among other screening modalities, the stool antigen test for *Hp* identifies active infection with excellent positive and negative predictive values. This test is recommended for diagnosis and monitoring of *Hp* infection eradication. Since 16 December 2022, this test has been reimbursed in France.

The stool antigen test is more reliable than serological tests in identifying active infection, it also provides rapid results, is non-invasive, and is easy to implement through simplified sample collection.

Quite recently, the excellent performances (Se 0.96, Sp 0.98) of non-invasive detection of *Hp* and its resistance to clarithromycin in stool by polymerase chain reaction (PCR) have been demonstrated [34]. This non-invasive test could modify and even improve the management of infected patients. These encouraging results open the prospect of performing this test in ambulatory care at the request of primary care physicians. It remains to be demonstrated that the use of this test in primary care will reduce the number of unnecessary endoscopies and allow specialists to be called in only for complex cases in which they are required.

Treatment modalities are well-codified and feasible in primary care. According to the latest recommendations, 14-day concomitant quadruple therapy is recommended as first-line treatment, combining a PPI, amoxicillin, clarithromycin, and metronidazole or quadruple therapy with bismuth for 10 days. However, the information provided by the different recommendations is insufficiently disseminated, even though Bucher et al. specified in 2017 that prevention is essential to the primary care provided in France by general practitioners [35]. These physicians say while they are highly involved in prevention, it still remains weak. Preventive procedures require improved information on how to deal, among other things, with *Hp* infection.

According to the World Organization of National Colleges, Academies and Academic Associations of General Practitioners (WONCA), health education and individual and community prevention are among the competencies required for the practice of general medicine [36].

Given their proximity to the general population and their coverage of the territory, GPs are ideally positioned to identify target patients eligible for *Hp* eradication in the context of gastric cancer prevention. Simplifying the recommendations available to GPs in their daily practice would optimize the identification of patients at risk and the management of *Hp* infection. It has been shown that a significant number of at-risk individuals are unscreened [16], including first-degree relatives of patients with gastric cancer, patients on long-term PPI therapy, and patients on long-term Non-steroidal anti-inflammatory drugs (NSAIDs) therapy.

In addition, increased *Hp* resistance to antibiotics, which has been apparent for several decades, requires improvement in the skills of prescribing physicians [16,33].

Increased prevention campaigns and educational programs for patients can help to optimize their management. Similarly, clear improvement in the transfer of information to general practitioners on prevention, screening, and the use of antibiotic therapy can help to improve the eradication of *Hp* and, consequently, reduce the incidence of gastric cancer.

In France, one of the steps taken in this direction was the creation in 2011 of a summary of recommendations by the French Study Group on *Helicobacter* (GEFH), designed to more closely coordinate management between general practitioners and gastroenterologists. This form should be attached directly to the digestive endoscopy report, whereas eradication and control protocols are overly complex.

Croze et al. conducted a retrospective and then a prospective study showing that this form significantly improved first-line treatment and the frequency and quality of eradication control prescribed by general practitioners [37]. The transmission of this recommendation form quadrupled the number of correctly treated and monitored patients [38]. 

At the Journées Francophones d’Hépato-gastroentérologie et d’Oncologie Digestive (JFHOD) in March 2020, Leclerc et al. presented the results of a follow-up analysis of practices between 2015 and 2018 on the diagnosis and treatment of *Hp* infection. This study follows the creation in May 2017 of two forms entitled “Relevance of care on the diagnosis and treatment of *Hp* infection in adults” by the French HAS and the National Professional Council of Hepato-Gastro-Enterology (CNP-HGE). Leclerc demonstrated not only improvement in the prescriptions of general practitioners, but also the need to continue the dissemination of the information contained in these forms.

The commercialization of several new high-performance non-invasive stool screening tests should revolutionize management of *Hp* infections. It will allow the application of a guided treatment by general practitioners in primary care [34,39].

Although carefully and rigorously conducted, the present review, which is narrative and non-systematic, cannot be considered free of limitations. First of all, and in accordance with INCA’s documentation procedures, only certain databases (PUBMED, Web of Science, BDSP, Open Grey) were included in the initial search, whereas other databases could have been additionally considered (Science Direct, EMBASE, Cochrane, etc.), which should be kept in mind by the reader. Such an approach has been privileged in order to quickly address the problem raised, without the need for additional (more time-consuming) research. For the same reason, PRISMA and MOOSE guidelines have not been completely applied to the present manuscript. In addition, it was decided to focus the present work on the analysis of adult and not pediatric infections (excluding articles dealing only with pediatric infection), considering that these were two distinct issues, and thereby increasing the comparability of the included studies. Furthermore, as the study was not designed for this purpose, no geographical variation in management could be identified in the present selected studies, with the exception of the screening method (serology in Europe and urea breath test in non-European countries).

With the exception of these limitations, this study provides important information on the issues raised (Table 1).

## 5. Conclusions

In conclusion, this study provides an update on the management of *Hp* infection in subjects under 40 years of age in primary care. Debate on the prospect of proactive involvement of primary care physicians in the control of *Hp* infection is consequently open. It could be hoped that the use of innovative non-invasive tests, which would identify its presence, and especially, its resistance to certain antibiotics, will simplify the practice of close-proximity physicians. The institutions of a country such as France are favorable to the development of research in primary care. Recently, institutional funding has been allocated for this purpose, and on the occasion of its 29th information day, the GEFH included general medicine among its activities. Informing, sensitizing, involving, supporting, and promoting the fight against *Hp* infection in primary care in a country such as France will be upcoming objectives. For example, the first French randomized study is underway [40]. To conclude, it is sufficient to quote the words of Pichon et al. in their editorial, “*It is time to establish new recommendations integrating the specificities of General Medicine as well as its main actor, the general practitioner, as leaders of this orientation*” [40].

## Figures and Tables

**Table 1:** Main information collected in the present review according to themes.

Theme	Main Information
Indications for screening for *Helicobacter pylori*	- In countries with a low prevalence of *Hp* infection (Europe, Canada, United States), population-based screening is not recommended. - It is recommended that patients under 45 years of age with no family history of gastric cancer and symptoms of dyspepsia without warning signs such as anemia, weight loss, dysphagia, palpable mass, and malabsorption be screened in primary care.
Screening and diagnostic methods: non-invasive tests	- Screening can be performed by a urea breath test, serology, and stool antigen detection in the primary care setting
Treatment modalities and knowledge of primary care physicians	- The last decade was characterized by a marked decline in the success of triple therapy due to the emergence of resistance to clarithromycin - In the absence of antibiotic susceptibility studies, treatment with concomitant quadruple therapy is recommended as first-line therapy, combining a PPI, amoxicillin, clarithromycin, and metronidazole for 14 days. - In case of previous use of macrolide or allergy to amoxicillin, a quadruple therapy with bismuth for 10 days is preferred, combining omeprazole with a bismuth salt, tetracycline, and metronidazole.

## Data Availability

Not applicable.

## Supplementary Materials

The following supporting information can be downloaded at: https://www.mdpi.com/article/10.3390/healthcare11030397/s1, Table S1: Standardized extraction grid of the selected articles; Table S2: Research equations. References [41,42,43,44,45].
