# Supplementary material for: Diagnostic and Therapeutic Management of Helicobacter pylori Infection in Primary Care: Perspective of Application in France and Narrative Review of the Literature
Source: Healthcare (Basel). 2023 Jan 30;11(3):397. doi: 10.3390/healthcare11030397 (PMC9914135; doi:10.3390/healthcare11030397)
Supplement: Supplementary file 1 [file healthcare-11-00397-s001.zip › healthcare-2156187-supplementary.pdf]

Supplementary Table S1. Standardized extraction grid of the selected articles.

Authors	Year	Title	Journal	Country	Objectives	Studied Population	Design	Ref
**Siddique O. *et al.***	*2018*	*Helicobacter pylori* infection: an update for the internist in the age of increasing global antibiotic resistance	The American journal of medicine	USA	Updated recommendations on the management of *Helicobacter pylori* infection for internists.	Update/Summary of Recommendations		[15]
**Seta T. *et al.***	2017	Effectiveness of *Helicobacter pylori* eradication in the prevention of primary gastric cancer in healthy asymptomatic people: a systematic review and meta-analysis comparing risk ratio with risk difference	PLOS One	Japan	To evaluate whether *Helicobacter pylori* eradication reduces the risk of gastric cancer by comparing the difference risk and relative risk.	Systematic review of the literature from 1965 to 2017		[41]
**Boltin D. *et al.***	2016	Attitudes and practice related to *Helicobacter pylori* infection among primary care physicians	European journal of gastroenterology et hepatology	Israel	To evaluate the practices of general practitioners in the management of *Helicobacter pylori* infection and to target corrective	General practitioners working for Clalit Health Services in Israel	Retrospective study by questionnaire over 10 days, 314 responses	[16]

					actions in areas where practices differ from the recommendations			
**Dore M.P. *et al.***	2016	Dyspepsia: when and how to test for *Helicobacter pylori* infection	Gastoenterology and Research practice	Italy	Summary of recommendations to guide the proper use of diagnostic tests and management of *Helicobacter pylori* in patients with dyspepsia by general practitioners.			[23]
**Fashner J. *et al.***	2015	Diagnosis and treatment of peptic ulcer disease and *H. pylori* infection	American Family Physician	USA	Updated recommendations for the management of *Helicobacter pylori* infection.	Literature review conducted in 2013		[24]
**Mégraud F. *et al.***	2014	Diagnosis of *Helicobacter pylori* infection	Helicobacter	France	Summary of new information on diagnostic methods for *Helicobacter pylori*.	Literature review		[19]
**Galban E. *et al.***	2012	Endoscopic findings and associated risk factors in primary health care settings in Havana, Cuba	Meddic review	Cuba	To describe the distribution frequencies of gastroduodenal diseases and search for associated risk factors.	Adults over 18 years of age who have not had FOGD in the previous year and who are seen in licensed health care centers in Havana	Prospective multicenter study from May to November 2007	[42]

**Nocon M. *et al.***	2009	Efficacy and cost effectiveness of the 13-C urea breath test as the primary diagnostic investigation for the detection of *Helicobacter pylori* infection compared to invasive and non-invasive diagnostic tests	GMS Health Technology Assessment	Germany	To investigate whether there are clinical and economic advantages to using the urea breath test for the diagnosis of *Helicobacter pylori* infection compared to other diagnostic methods.	Adult patients over 18 years of age with symptoms of unexplored dyspepsia.	Systematic Review of the literature	[28]
**Tepes B.**	2009	Can gastric cancer be prevented?	Journal of physiology and pharmacology	Slovenia	Prevention of gastric cancer through the management of *Helicobacter pylori* infection, recognized as a risk factor.	Literature review		[18]
**Cardin F. *et al.***	2007	Implementation of a guideline versus use of individual prognostic factors to prioritize waiting lists for upper gastrointestinal endoscopy	European Journal of gastro enterology and hepatology	Italy	Evaluate whether it is more appropriate to prioritize endoscopy waiting lists based on simple, easily detectable criteria or whether applying European recommendations	Adult patients over 18 years of age admitted to the outpatient department for FOGD for dyspepsia in the Local Health District of Padova	Prospective study from October 2000 to April 2001	[43]

					would increase the number of positive FOGD			
**Shirin H. *et al.***	2004	Application of Maastricht 2-2000 Guidelines for the management of *Helicobacter pylori* among specialists and primary care physicians in Israel	Journal of clinical gastroenterology	*Israel*	To assess the impact of the Maastricht II recommendations on the knowledge, practices, and management of *Helicobacter pylori* infection among general practitioners, internists, and gastroenterologists.	General practitioners working in clinics, gastroenterologists and internists working in hospitals.	Prospective study from December 2001 to June 2002. Questionnaires submitted to general practitioners and specialists.	[33]
**Sullivan T. *et al.***	2004	*Helicobacter pylori* and the prevention of gastric cancer	Canadian journal of gastroenterology	Canada	State of the art of *Helicobacter pylori* treatment and gastric cancer prevention and identification of critical areas to guide educational approaches for health professionals and patients.	Meeting of a panel of twelve experts in various fields by Cancer Care Ontario	Expert group meeting (October 2002)	[20]

**Huang J. *et al.***	2003	Has education about *Helicobacter pylori* infection been effective? Worldwide survey of primary care physicians	Journal of gastroenterology and hepatology	Asia, Occitania, Europe, South Africa, USA, Canada, South America	Review of global knowledge on the management of *Helicobacter pylori* infection to understand the differences in management between countries and identify areas where educational action may be needed.	470 General Practitioners from 29 countries.	Prospective study from January 2000 to August 2000. Questionnaire by telephone survey.	[22]
**Perri F. *et al.***	2002	Appropriateness of urea breath test: a prospective observational study based on Maastricht 2000 guidelines	Alimentary, pharmacology and Therapeutics	Italy	To evaluate the indication for urea breath testing and the appropriateness of its prescription according to recommendations by general practitioners and gastroenterologists in the diagnosis and post-treatment confirmatory testing	1320 patients from the gastroenterology unit of the hospital of San Giovani Rotondo	One-year prospective observational study in 2001	[25]

					of *Helicobacter pylori* infection.		
**Roberts A.P. *et al.***	2000	The management of *Helicobacter pylori* infection in primary care: a systematic review of the literature	Family practice	UK, Denmark and the Netherlands	To evaluate the role of Helicobacter pylori in upper gastrointestinal diseases (GERD, gastric cancer, ulcer) and to review the medico-economic management of the infection (diagnostic methods, treatments, costs).	Systematic review of the literature (June 1966 to June 1999)	[44]
**Deltenre M.**	2000	La dyspepsie en médecine générale : approche diagnostique actualisée	Revue Médicale de Bruxelles, Gastro entérologie.	Belgium	Update on the management of dyspepsia in general practice.	Literature review	[27]
**Fendrick A.M.**	2000	The role of economic evaluation in the diagnosis and treatment of *Helicobacter pylori* infection	Gastroenterology Clinics of North America	USA	To help clinicians identify which patients would benefit clinically from *Helicobacter pylor*i eradication and determine the	Publication of expert opinion	[45]

					cost of strategies for diagnosis, treatment and follow-up.			
**Malfertheiner P.**	1999	The Maastricht recommendations and their impact on general practice	European Journal of gastroenterology and hepatology	The Netherlands	To assess the impact of the Maastricht recommendations among general practitioners and gastroenterologists.	General practitioners and gastroenterologists	Meeting of general practitioners and gastroenterologists from 19 European countries	[7]
**Wu J. *et al.***	1999	Treatment of *Helicobacter pylori* infection	Hong Kong Medicine Journal	China	To clarify the therapeutic management of *Helicobacter pylori* infection.	Literature review		[30]
**Tytgat G.N.J.**	1998	Review article: practical management issues for the *Helicobacter pylori* infected patient at risk of gastric cancer	Alimentary, Pharmacology and Therapeutics	The Netherlands	Update on the management of *Helicobacter pylori* infection in patients at risk for gastric cancer	Literature review		[17]
**Stanghellini V. *et al.***	1997	Widespread eradication of *Helicobacter pylori*: a debate	Helicobacter	Italy	Debate on the value of screening and treatment of *Helicobacter pylori* in the general population.	A critical review of the literature		[21]

**Bleau BL. *et al.***	1995	*Helicobacter pylori*: an update for primary care physicians	Comprehensive Therapy	USA	Evaluation of the involvement of *Helicobacter pylori* in the development of upper gastrointestinal diseases (gastritis, ulcers, gastric cancers and lymphomas).	Publication of expert opinion	[29]

Supplementary Table S2. Research equations.

**Research site**	**Research equations**	**Results**
**Pubmed**	**R1 :** (cancer[tiab] OR neoplas*[tiab] OR tumor[tiab] OR tumors[tiab] OR tumour[tiab] OR tumours[tiab] OR malign*[tiab] OR adenocarcinoma*[tiab] OR carcinoma*[tiab]) AND (stomach[tiab] OR gastric[tiab]) AND ("Helicobacter pylori"[tiab] OR "H. pylori "[tiab] OR "*HP*"[tiab]) AND (treatment*[tiab] OR therap*[tiab] OR management[tiab] OR eradication*[tiab] OR immunotherap*[tiab] OR vaccin*[tiab] OR detection[tiab] OR search*[tiab] OR endoscopy[tiab] OR fibroscopy[tiab] OR "urease activity" [tiab] OR biopsy[tiab] OR "urea breath test" [tiab] OR "stool antigen test"[tiab]) AND (prevention[tiab] OR prophylactic[tiab] OR prophylaxis[tiab]) AND (general medicine[tiab] OR general practi*[tiab] OR family practi*[tiab] OR primary care[tiab]) AND (indication*[tiab] OR recommandation*[tiab] OR recommendation*[tiab] OR guideline*[tiab]) AND hasabstract[text] AND ("1982/01/01"[PDAT] : "2019/01/24"[PDAT]) AND (English[lang] OR French[lang])	2
	**R2 :** (cancer[tiab] OR neoplas*[tiab] OR tumor[tiab] OR tumors[tiab] OR tumour[tiab] OR tumours[tiab] OR malign*[tiab] OR adenocarcinoma*[tiab] OR carcinoma*[tiab]) AND (stomach[tiab] OR gastric[tiab]) AND ("Helicobacter pylori"[tiab] OR "H. pylori "[tiab] OR "*HP*"[tiab]) AND (treatment*[tiab] OR therap*[tiab] OR management[tiab] OR eradication[tiab] OR immunotherap*[tiab] OR vaccin*[tiab] OR detection[tiab] OR search*[tiab] OR endoscopy[tiab] OR fibroscopy[tiab] OR "urease activity"[tiab] OR biopsy[tiab] OR "urea breath test"[tiab] OR "stool antigen test"[tiab]) AND (general medicine[tiab] OR general practi*[tiab] OR family practi*[tiab] OR primary care[tiab]) AND (indication*[tiab] OR recommandation*[tiab] OR recommendation*[tiab] OR guideline*[tiab]) AND hasabstract[text] AND ("1982/01/01"[PDAT] : "2019/01/24"[PDAT]) AND (English[lang] OR French[lang])	25
	**R3 :** (cancer[tiab] OR neoplas*[tiab] OR tumor[tiab] OR tumors[tiab] OR tumour[tiab] OR tumours[tiab] OR malign*[tiab] OR adenocarcinoma*[tiab] OR carcinoma*[tiab]) AND (stomach[tiab] OR gastric[tiab]) AND ("Helicobacter pylori"[tiab] OR "H. pylori "[tiab] OR "*HP*"[tiab]) AND (treatment*[tiab] OR therap*[tiab] OR	65

	management[tiab] OR eradication*[tiab] OR immunotherap*[tiab] OR vaccin*[tiab] OR detection[tiab] OR search*[tiab] OR endoscopy[tiab] OR fibroscopy[tiab] OR "urease activity" [tiab] OR biopsy[tiab] OR "urea breath test" [tiab] OR "stool antigen test"[tiab]) AND (prevention[tiab] OR prophylactic[tiab] OR prophylaxis[tiab]) AND (indication*[tiab] OR recommandation*[tiab] OR recommendation*[tiab] OR guideline*[tiab]) AND hasabstract[text] AND ("1982/01/01"[PDAT] : "2019/01/24"[PDAT]) AND (English[lang] OR French[lang])	
	**R4 :** (cancer[tiab] OR neoplas*[tiab] OR tumor[tiab] OR tumors[tiab] OR tumour[tiab] OR tumours[tiab] OR malign*[tiab] OR adenocarcinoma*[tiab] OR carcinoma*[tiab]) AND (stomach[tiab] OR gastric[tiab]) AND ("Helicobacter pylori"[tiab] OR "H. pylori "[tiab] OR "*HP*"[tiab]) AND (treatment*[tiab] OR therap*[tiab] OR management[tiab] OR eradication[tiab] OR immunotherap*[tiab] OR vaccin*[tiab] OR detection[tiab] OR search*[tiab] OR endoscopy[tiab] OR fibroscopy[tiab] OR "urease activity"[tiab] OR biopsy[tiab] OR "urea breath test"[tiab] OR "stool antigen test"[tiab]) AND (indication*[tiab] OR recommandation*[tiab] OR recommendation*[tiab] OR guideline*[tiab]) AND hasabstract[text] AND ("1982/01/01"[PDAT] : "2019/01/24"[PDAT]) AND (English[lang] OR French[lang])	358
	**R5** : ("Helicobacter Infections"[Mesh] OR "Helicobacter pylori"[Majr]) AND ("Stomach Neoplasms/prevention and control"[Mesh]) AND prevent*[tiab] AND ("1982/01/01"[PDAT] : "2019/01/24"[PDAT])	337
**Web of Science**	TS=((cancer OR neoplas* OR tumor OR tumors OR tumour OR tumours OR malign* OR adenocarcinoma* OR carcinoma*) AND (stomach OR gastric) AND ("Helicobacter pylori" OR "H. pylori" OR "*HP*") AND (treatment* OR therap* OR eradication OR immunotherapy* OR vaccin* OR detection OR search* OR endoscopy OR fibroscopy OR "urease activity" OR biopsy OR "urea breath test" OR "stool antigen test") AND (prevention OR prophylactic OR prophylaxis) AND (indication* OR recommandation* OR*recommendation* OR guideline*))	94
**BDSP**	Mcl=([helicobacter pylori] [cancer] [estomac] [prevention]	13
**Open grey**	(cancer OR neoplas OR tumor OR tumors OR tumour OR tumours OR malign OR adenocarcinoma OR carcinoma) AND (stomach OR gastric) AND ("Helicobacter pylori" OR "H. pylori " OR "*HP*") AND (treatment OR therap OR eradication OR immunotherapy OR vaccin OR detection OR search OR endoscopy OR fibroscopy) AND (prevention OR prophylactic OR prophylaxis) AND (indication OR recommandations OR recommendations OR guidelines)	0
